# Application of V_2_O_5_/WO_3_/TiO_2_ for Resistive-Type SO_2_ Sensors

**DOI:** 10.3390/s110302982

**Published:** 2011-03-07

**Authors:** Noriya Izu, Gunter Hagen, Daniela Schönauer, Ulla Röder-Roith, Ralf Moos

**Affiliations:** 1 Functional Materials Laboratory, University of Bayreuth, Universitätsstraße 30, 95440 Bayreuth, Germany; E-Mails: noriya.izu@uni-bayreuth.de (N.I.); gunter.hagen@uni-bayreuth.de (G.H.); daniela.schoenauer@uni-bayreuth.de (D.S.); ulla.roeder@uni-bayreuth.de (U.R.-R.); 2 National Institute of Advanced Industrial Science and Technology (AIST), Advanced Manufacturing Research Institute, 2266-98 Anagahora, Shimo-Shidami, Moriyama-ku, Nagoya 463-8560, Japan

**Keywords:** sulfur dioxide, selective catalytic reduction (SCR), gas sensor, vanadia tungsten titania

## Abstract

A study on the application of V_2_O_5_/WO_3_/TiO_2_ (VWT) as the sensitive material for resistive-type SO_2_ sensor was conducted, based on the fact that VWT is a well-known catalyst material for good selective catalytic nitrogen oxide reduction with a proven excellent durability in exhaust gases. The sensors fabricated in this study are planar ones with interdigitated electrodes of Au or Pt. The vanadium content of the utilized VWT is 1.5 or 3.0 wt%. The resistance of VWT decreases with an increasing SO_2_ concentration in the range from 20 ppm to 5,000 ppm. The best sensor response to SO_2_ occurs at 400 °C using Au electrodes. The sensor response value is independent on the amount of added vanadium but dependent on the electrode materials at 400 °C. These results are discussed and a sensing mechanism is discussed.

## 1. Introduction

Today, coal is an important fossil fuel, that will continue to play an important role through the 21st century [1], in addition to renewable energies. The identified coal reserves are expected to last for 150 years, in contrast to oil and natural gas reserves which may last for only 41 and 65 years, respectively. The cost of coal is about 1/6 that of oil and natural gas. Therefore, it is predicted that coal consumption will increase by 2.5% per year between 2003 to 2030 [1]. Coal, however, faces significant environmental challenges, since exhaust gas from coal combustion contains limited emission substances such as SO_2_, NO*_x_*, *etc.* [1]. For instance, the exhaust gas from coal-fired kilns includes 300–500 ppm of SO_2_, and flue gases or stack gas include 500–4,000 ppm and 5–10 % of SO_2_, respectively [2].

For SO_2_ monitoring and control, SO_2_ sensors with a good durability at high SO_2_ concentrations and at high temperatures are needed. Due to the stability requirements, we focused on sensors based on V_2_O_5_/WO_3_/TiO_2_ (abbreviated VWT), because VWT is a well-known material for selective NO_x_ catalytic reduction catalysts using ammonia as a reducing agent (NH_3_-SCR) [3–5]. It is applied, for instance, for NO_x_ removal in coal power plants or for DeNOx processes in diesel exhausts. VWT shows a good stability in sulfur oxide-rich combustion exhausts [5]. It has already been suggested as a sensor material for ammonia sensing purposes in exhausts [6–8].

There are only a few papers reporting on SO_2_ sensors utilizing V_2_O_5_/TiO_2_ (without WO_3_) [9,10] and there is no paper that involves VWT as an SO_2_ sensitive material, although there are many papers about SO_2_ oxidation of VWT in SCR catalysts [11–16]. Morris and Egdell reported on a resistive bulk-type sensor of small V_2_O_5_-TiO_2_ bars with Pt electrodes [10]. In contrast, in our study, we realized a planar setup for our resistive sensors and VWT with a V_2_O_5_ concentration of 1.5 wt% and 3.0 wt% served as the sensitive functional material. We varied the electrode materials between Pt and Au and measured the resistance changes when exposed to SO_2_ and/or other gases. Unexpectedly, it was found out that for the SO_2_ response the electrode materials play a more crucial role than the vanadium concentration. Furthermore, we discuss the sensing mechanism in this study. It should be noted here that the major objective of this study is to investigate whether commercially available and proven long-term stable SCR catalyst materials are suitable as materials for resistive SO_2_ sensors. Material development is not a subject of this study.

## 2. Experimental

Typical alumina substrates for thick-film technique (96% Al_2_O_3_) were prepared. On the front side of the alumina substrate, Pt or Au interdigital electrodes with a line and space definition of 100 μm were screen-printed and fired, and a platinum heater was screen-printed on the reverse side and fired. Then, the sensor layers were fabricated. V_2_O_5_/WO_3_/TiO_2_ (VWT) was used as a sensing material. VWT powders were supplied by Argillon GmbH. The V_2_O_5_ concentration was 1.5 or 3.0 wt% and the WO_3_ concentration was *ca.* 8–10 wt%. In this study, *x*%VWT is an abbreviation that stands for the composition *x* wt% V_2_O_5_/8–10 wt% WO_3_/TiO_2_, in which *x* is 1.5 or 3.0. The VWT powders were mixed with an organic binder to prepare pastes suitable for screen-printing. Afterwards, the pastes were screen-printed on the alumina substrates with the electrodes and fired at 700 °C for 20 min. The VWT powder or layer was characterized by scanning electron microscopy (SEM, Leo1450VP), powder X-ray diffraction (PANalytical X’Pert Pro, Cu Kα radiation), and contact probe profilometer (MarSurf). The thickness of VWT layers is in the range of 10–15 μm.

The sensors were heated by the heater on the reverse side when the sensing properties were determined. The temperature was controlled by utilizing the relationship between heater resistance (as determined in four-wire-technique) and temperature, which had been investigated before using an infrared pyrometer. By this method, the sensing element is heated correctly without the influence of gas flow ratio.

To investigate its sensing properties, the sensor devices were placed into a test chamber with an angle of incidence parallel to the gas flow. The resistance between the electrodes (*i.e.*, the sensor output), was measured by a two-wire-method with a digital multimeter (Keithley 2700 series). The total gas flow amounted to either 1 or 5 L/min. We confirmed that the sensor resistance in base gas was independent of the total gas flow rate. Compressed air was used as a base gas to measure the response to SO_2_, while a gas mixture of 20% O_2_ and N_2_ was used in the case of the response measurements towards other gases like CO, CO_2_, C_3_H_8_, H_2_O, H_2_, NO, and NH_3_. The water concentration of the compressed air was checked by FTIR to be below 0.5%. Since the other gases are supplied from gas cylinders, the water concentration of them is less than 0.1%. We defined the response value (*S*) in accordance to Equation (1):

(1) $$S=(R_{0}-R)/R_{0}$$

where *R*_0_ denotes the resistance measured in base gas and *R* denotes the resistance in presence of the analyte.

## 3. Results and Discussion

**Figure 1.**
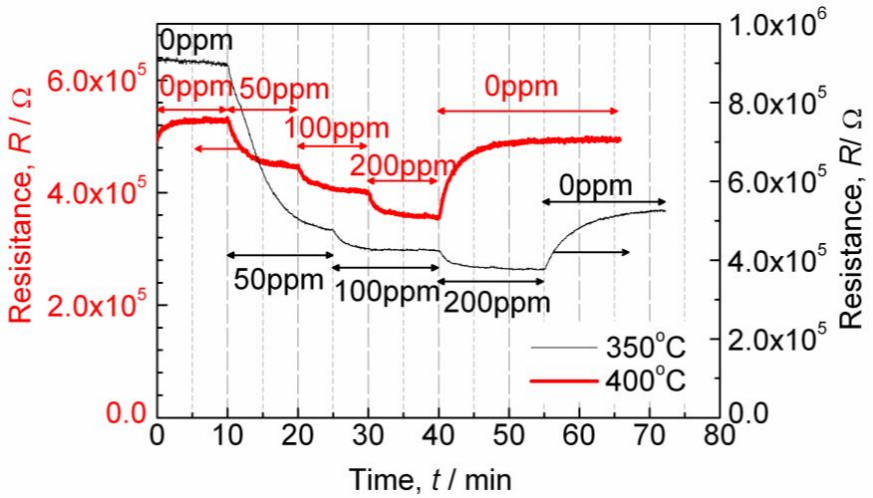
Typical behavior of the sensor resistance when SO_2_ is added stepwise to the base gas. Sensor: 3.0%VWT; Au electrodes; further details see text; *T* = 350 °C and *T* = 400 °C.

The powder X-ray diffraction revealed that titanium oxide exists in our VWT as an anatase phase, even after annealing at 700 °C. The thick-films have a porous structure. First of all, the sensor resistance response towards SO_2_ was investigated. As a base gas, compressed air was used, so that a small amount of water vapor was included in the gas. Figure 1 shows a typical sensor response behavior for a sensor of 3.0%VWT and Au electrodes when SO_2_ is added.

At 400 °C, the resistance decreases as soon as SO_2_ is admixed. After the decrease, the resistance is almost constant with a change of less than 1.5% within 5 min. The resistance decreases with increasing SO_2_ concentrations. After switching back to base gas (without SO_2_), the resistance returns approximately to its initial value (within the difference of about 5%) after SO_2_ exposure, which can be considered as almost constant with time at 400 °C or more. In that case, the initial values in the base gas were used as *R*_0_. At 350 °C, however, the resistance decreased largely when 50 ppm SO_2_ was added. After switching back to SO_2_-free base gas, the resistance did not return to its initial value. That is to say, a base line shift was observed. Therefore, at 350 °C the resistance in the base gas after SO_2_-exposure was used as *R*_0_.

**Figure 2.**
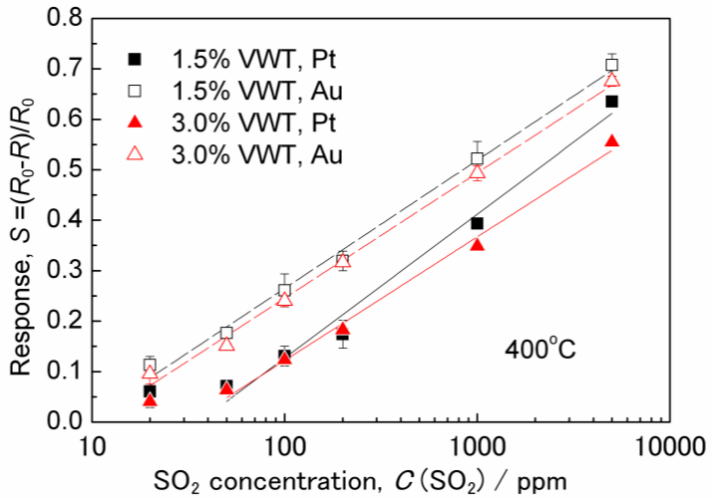
Relationship between the response value, *S*, of the sensor resistance and the SO_2_ concentration at *T* = 400 °C.

Figure 2 shows the relationship between the sensor response value, *S*, and the SO_2_ concentration. The ordinate is plotted in a linear scale, whereas a logarithm scale is used for the SO_2_ concentration on the abscissa. The response value, *S*, increases with the SO_2_ concentration for all samples. The responses values for sensors with Au electrodes are larger than those for sensors with Pt electrodes. The responses are at a first order independent on the amount of added V_2_O_5_. For the sensors with Au electrodes, a linear relationship (in a semilogarithmic representation) can be observed in an SO_2_ concentration range from 20 to 5,000 ppm. At low SO_2_ concentrations, the response values with Pt electrodes do not depend on the SO_2_ concentration anymore.

Different graphs like log *S vs.* log *c*(SO_2_), log (*R/R*_0_) *vs.* log *c*(SO_2_), *R*_0_*/R vs.* log *c*(SO_2_), log (*R*_0_*/R*) *vs.* log *c*(SO_2_), and *R*_0_*/R vs. c*(SO_2_) were plotted using the data of Figure 2. However, none of the graphs yielded a linear relationship. This agrees with findings of Morris and Egdell, who reported a non-linear relationship of *S vs. c*(SO_2_) [10]. We re-plotted their data on an *S vs.* log *c*(SO_2_) graph and found also a linear relationship between *S* and log *c*(SO_2_). Hence, the data obtained from our planar sensor setup are consistent with reference [10]. Up to now, we have not been able to clarify the physico-chemical reason for the linear relationship in the *S vs.* log *c*(SO_2_) graph, but the finding of a linear relationship could be useful for practical sensor applications.

**Figure 3.**
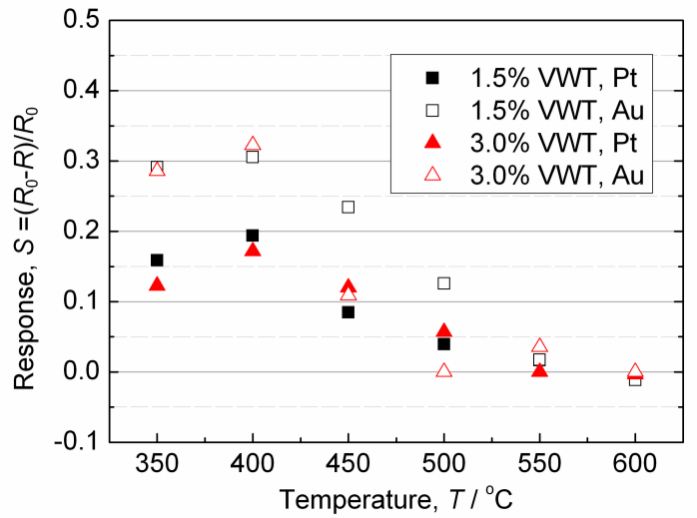
Relationship between the response value, *S*, of the sensor resistance to 200 ppm SO_2_ and the operating temperature.

Figure 3 shows how the response value, *S*, towards 200 ppm SO_2_ varies with the operating temperature. Over 400 °C, the response values decrease with increasing temperature. At 400 °C, a maximum sensor response occurs for each sensor. This behavior is similar to the result reported in reference [10]. In Figure 3, the response values almost do not depend on the amount of added V_2_O_5_ at 400 °C or less, while the response values for the sensor with Au electrodes are larger than those for the sensor with Pt electrodes. As shown in Figures 2 and 3, the responses are almost independent on the V_2_O_5_ content, but dependent on the electrode materials at 350 and 400 °C, the temperature range in which the sensors respond strongly, especially with Au electrodes. Therefore, the interface between VWT and the electrodes seems to be important for the response towards SO_2_.

**Figure 4.**
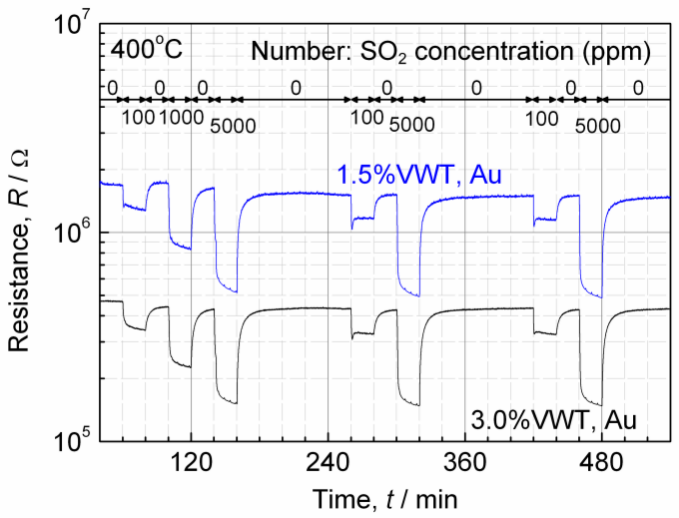
Repeated response for the sensors with Au electrodes at 400 °C.

The reproducibility was investigated for the sensors at 400 °C. The error bars in Figure 2 show the standard deviation of the data. In 100 ppm SO_2_, the coefficient of variation (CV), which is defined as the ratio of the standard deviation to the mean, of “*S*” was calculated. The CV for the sensor with Au electrodes and 3.0%VWT is 5% and the CVs for the others are 12–15%. Figure 4 shows the sensor resistance, when sensors with Au electrodes are repeatedly exposed to varying SO_2_ concentrations of 100 ppm and 5,000 ppm. Although the base line decreases 10% at first, it does not change after 200 min in Figure 4 (within the difference of about 5%). This result indicates that aging processes may be effective for stabilizing the base line. However, the responses to repeated SO_2_ pulses were almost identical (Figure 4). These results confirm the reproducibility. Furthermore, we also confirmed the process reproducibility by checking the response of other sensors prepared with the same parameters.

**Figure 5.**
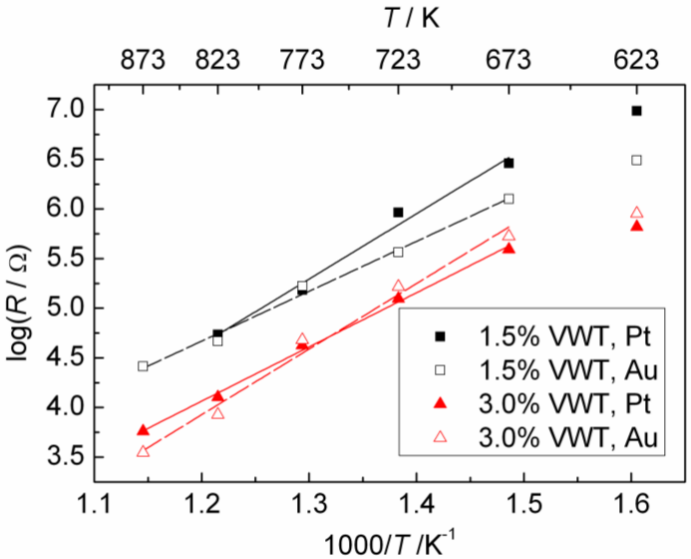
Arrhenius-type representation of the sensor resistance in compressed air (without SO_2_).

Figure 5 shows the relationship between the sensor resistance and the temperature in an Arrhenius-type representation. In order to determine the resistivity or the conductivity, the film thickness and the porosity of each single thick film should be determined precisely. This is difficult to do with a sufficient accuracy. Therefore, we plotted only the resistance, *R*, and not the resistivity on the ordinate. The temperature dependency, however, should be the same. In Figure 5, the slope in the log *R vs.* 1/*T* —representation changes at around 400 °C, and the slope over 400 °C becomes larger. Reference [17] reported the resistances of 2%VWT in the range of 180 °C to 300 °C. The activation energy calculated from their data is approximately 0.7 eV, which is smaller than the one here-determined (*ca.* 1 to 1.3 eV) over 400 °C. Therefore, the slope under 400 °C is expected to be less than 1 eV, if the measurement is carried out in the range of 180 °C to 300 °C. The changing slope around 400 °C might indicate that the mechanism of the electrical conductivity varies at this temperature. This may be related to the maximum sensor response at around 400 °C.

**Figure 6.**
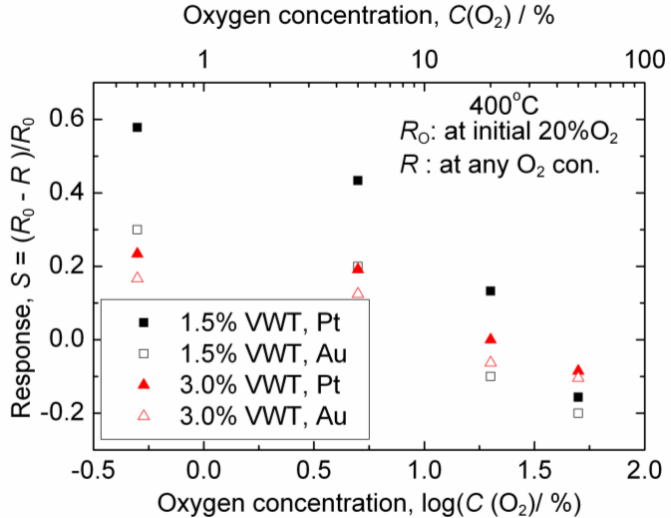
Oxygen concentration dependence of the sensor resistance at *T* = 400 °C.

Since the oxygen concentration may vary in combustion exhausts, an optimal sensor signal should not be affected by oxygen fluctuations. Figure 6 shows the oxygen concentration dependency of the resistance. In this special experiment, the oxygen concentration was controlled by mixing dry O_2_ and dry N_2_ gas supplied from gas cylinders. Therefore, the water vapor concentration in the mixture is much smaller (nominally zero) than that in compressed air. In all cases, the oxygen dependency in the low concentration range is smaller than that in the high range. In combustion exhaust gases, the oxygen concentration is usually below 5% so that the sensors manufactured for this study may be applicable for practical use. The sensors with Au electrodes and 3.0%VWT exhibit a smaller oxygen dependency than the devices with Pt electrodes and 1.5%VWT, respectively.

**Figure 7.**
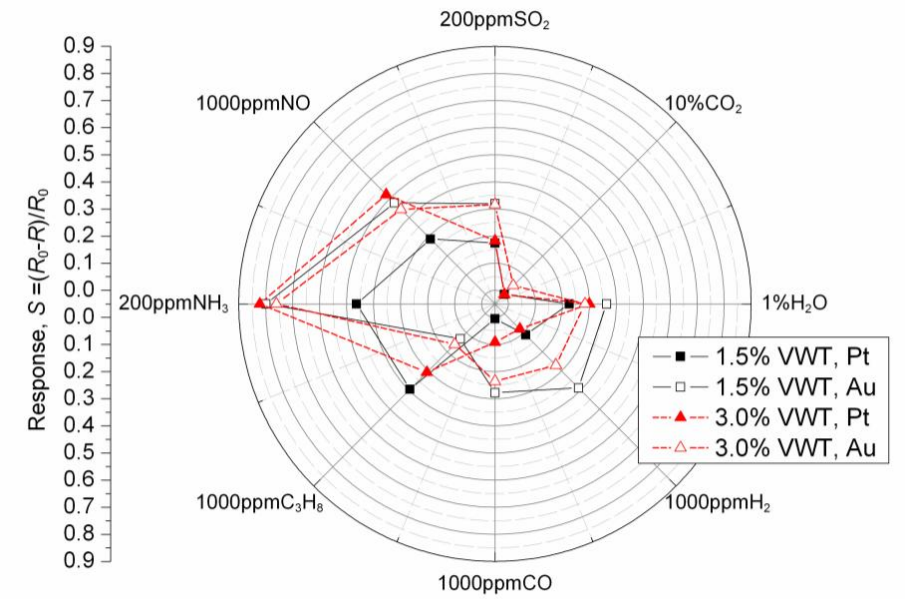
Responses against to the various gases at *T* = 400 °C.

Figure 7 summarizes the sensor response values towards various gases in a radar plot. The base gas used in this experiment was 20% O_2_ in N_2_. Again, the gas composition was mixed with mass flow controllers from O_2_ and N_2_ supplied from gas cylinders. Therefore, the water vapor concentration in the mixture was much smaller (almost zero) than that in compressed air.

None of the sensors responds significantly to an addition of 10% CO_2_ to the base gas. The response values, *S*, of all sensors for 1% H_2_O and those for 1,000 ppm NO are almost the same and a little larger compared to those for 200 ppm SO_2_, respectively. The response values of the sensors with Au electrodes for 1,000 ppm H_2_ are almost the same as those for 200 ppm SO_2_, while the responses value of the others are smaller than those for 200 ppm SO_2_. The response values of all sensors for 200 ppm NH_3_ are twice as large as those for 200 ppm SO_2_, so that it is reconfirmed that VWT resistive sensors show a good response to NH_3_ [18]. The response value ratio of 1,000 ppm CO to 1,000 ppm C_3_H_8_ is much different between Au and Pt electrodes. In the case of Au electrodes, the response value for 1,000 ppm CO is larger than that for 1,000 ppm C_3_H_8_, while the response value for 1,000 ppm CO is smaller than that for 1,000 ppm C_3_H_8_ in the case of Pt electrodes. Although the sensors investigated in this study respond markedly to NH_3_, the application may be restricted only for some cases. Especially when applied in a coal combustion exhaust, almost no ammonia is present. However, NO might be a more critical component, which need to be compensated or removed by a filter; some zeolites may be a good material for that purpose [19]. With the exception of NO, the selectivity should be high enough for a practical application of the VWT-based sensor for SO_2_ monitoring or controlling purposes.

In this section, the SO_2_ sensing mechanism is discussed in the light of the above-presented results and some selected literature from both the sensor and the catalysis community. It should be annotated here that our proposed model is just a suggestion, but is supported by the literature of both scientific communities. Dunn *et al.* [20] proposed a mechanism for sulfur dioxide oxidation over solid vanadia catalyst. Based on it, we suggest the sensor to behave as the following: SO_2_ oxidation to SO_3_ occurs on V_2_O_5_. In parallel, the valence of the V^5+^ ions change to V^3+^ [20]:

(2) $${\text{SO}}_{2}+V^{5+}-O-↔{\text{SO}}_{3}+V^{3+}$$

In Equation 2, -O- denotes oxide ions in vanadium-oxygen-support bond [20]. In the next step, V^3+^ ions react either with oxygen molecules (½ O_2_(g)) or with adsorbed oxygen species on the surface (O_ads_), whereby V^5+^ -O- forms [20]:

(3) $$V^{3+}+(½O_{2}(g)\;\text{or}\;O_{\text{ads}})↔V^{5+}-O-$$

When O_2_(g) or O_ads_ in Equation 3 are supplied from TiO_2_, charged point defects (V_O_¨: oxide ion vacancies, Ti_i_¨: interstitial Ti ions) and conduction electrons (e’) form in TiO_2_ according to the following equations [21,22]:

(4) $$O_{O}{}^{x}↔V_{O}¨+2e'+(½O_{2}(g)\;\text{or }O_{\text{ads}})\;\text{or }{\text{TiO}}_{2}↔{\text{Ti}}_{i}¨+2e'+(½O_{2}(g)\;\text{or}\;O_{\text{ads}})$$

It is under discussion whether the newly formed point defects in anatase titania are oxygen vacancies [21] or interstitial Ti ions, which according to [22], are not completely ionized. However, for the discussion of the sensing mechanism, this question is secondary, since the formation of both defects in TiO_2_ lead to a simultaneous formation of mobile electrons [17,21,22]. Due to this electron density increase, the resistance decreases.

Oxygen ions (O^−^_ads_) adsorb on the surface of WO_3_ [23]. Then, when O^−^_ads_ on the surface of WO_3_ moves to vanadium oxide according to Equation 5, a conduction electron forms:

(5) $$O^{-}{}_{\text{ads}}↔O_{\text{ads}}+e'$$

This leads to a resistance decrease as well. The formed O_ads_ reacts with V^3+^ based on Equation 3. Then, based on Equation 2, SO_2_ is oxidized to SO_3_. The mechanism mentioned above cannot explain the reason why the response value, *S*, depends on the electrode materials. Gerlich *et al.* [24] reported that the resistance change at the interface between a specific oxide and an electrode is larger than the change of the intrinsic resistance of the oxide when the oxide is used as a gas sensing material. Therefore, for the sensor manufactured in this study, the interface between VWT and electrodes might play a key role. However, the details have not been clarified yet.

Although it is assumed that SO_2_ is oxidized on vanadium oxide as discussed above, it is possible to assume that the following reactions occur: SO_2_ reduces TiO_2_ directly and/or SO_2_ reacts with adsorbed oxide ions (O^−^_ads_) on WO_3_. Furthermore, at 450 °C and 500 °C, *S* seems to be only slightly affected by the electrode materials. In this temperature range, different reactions may occur. Unfortunately, the literature considers VWT especially due to its catalytic properties. It has therefore a strong focus on lower temperature, at which the SCR reactions predominate. The high temperature range is often neglected since it is not of high interest for NO_x_ removal applications. Therefore, in future work, the detailed sensing mechanism should be investigated by other experiments and analyses, with a special focus of temperatures of 400 °C or more.

## 4. Conclusions

Planar resistive SO_2_ sensors based on VWT as a sensitive functional material that is very stable in exhausts were manufactured and their sensing properties were investigated in this study. The obtained results are as follows: the resistance of VWT decreases with increasing SO_2_ concentration. The relationship between the response value (*S* = (*R*_0_ − *R*)/*R*_0_) and SO_2_ concentration is linear in a representation of *S vs.* the logarithm of the SO_2_ concentration. The best response value occurs at 400 °C in the case of Au electrodes. The response value does hardly depend on the vanadium content but strongly on the electrode materials at 400 °C. From these results, some sensing mechanisms were discussed. From an application point of view, the selectivity needs to be improved in the future, especially with respect to nitrogen oxides. An optimization of the electrode materials or an addition of noble metal particles on the VWT surface may be an appropriate solution, besides the application of filter layers. An optimization of WO_3_ concentration may be considered as well.
